# The Smallpox Situation

**Published:** 1911-07

**Authors:** 


### The Smallpox Situation

THE Health Department of Buffalo announces that during the latter part of May and the early part of June there were 25 cases of smallpox in the city. In every case careful and painstaking search was made for possible spread and histories were taken with minute detail.

An illuminating fact and one that stands unanswerable by that associated cult of the antivivisectionists, the antivaccinationists, is that in every case of smallpox reported the individual afflicted with the disease had never been vaccinated. The situation was at no time serious; there was no moment when the health department did not have it in hand and it speaks well for efficiency that the miniature epidemic was stamped out before it was well begun.

